# TrieDedup: a fast trie-based deduplication algorithm to handle ambiguous bases in high-throughput sequencing

**DOI:** 10.1186/s12859-024-05775-w

**Published:** 2024-04-18

**Authors:** Jianqiao Hu, Sai Luo, Ming Tian, Adam Yongxin Ye

**Affiliations:** 1 Program in Cellular and Molecular Medicine, Boston Children’s Hospital, Boston, MA USA; 2 Present Address: Department of Biology, University of Washington, Seattle, WA USA; 3 Harvard Medical School, Boston, MA USA; 4 Howard Hughes Medical Institute, Boston Children’s Hospital, Boston, MA USA; 5 Present Address: School of Basic Medical Science, Tsinghua University, Beijing, China

**Keywords:** Deduplication, Ambiguous bases, Trie, Prefix tree, Next-generation sequencing

## Abstract

**Background:**

High-throughput sequencing is a powerful tool that is extensively applied in biological studies. However, sequencers may produce low-quality bases, leading to ambiguous bases, ‘N’s. PCR duplicates introduced in library preparation are conventionally removed in genomics studies, and several deduplication tools have been developed for this purpose. Two identical reads may appear different due to ambiguous bases and the existing tools cannot address ‘N’s correctly or efficiently.

**Results:**

Here we proposed and implemented TrieDedup, which uses the trie (prefix tree) data structure to compare and store sequences. TrieDedup can handle ambiguous base ‘N’s, and efficiently deduplicate at the level of raw sequences. We also reduced its memory usage by approximately 20% by implementing restrictedDict in Python. We benchmarked the performance of the algorithm and showed that TrieDedup can deduplicate reads up to 270-fold faster than pairwise comparison at a cost of 32-fold higher memory usage.

**Conclusions:**

The TrieDedup algorithm may facilitate PCR deduplication, barcode or UMI assignment, and repertoire diversity analysis of large-scale high-throughput sequencing datasets with its ultra-fast algorithm that can account for ambiguous bases due to sequencing errors.

**Supplementary Information:**

The online version contains supplementary material available at 10.1186/s12859-024-05775-w.

## Background

High-throughput sequencing methods have been adapted and applied in many fields of biological studies, including immune repertoire studies [1, 2]. Polymerase chain reaction (PCR), used during high-throughput sequencing library preparation, may lead to overrepresented templates, when multiple copies of the same DNA templates are amplified. These identical reads are termed as PCR duplicates [3]. PCR amplification may be biased based on the sequence and quantity of DNA templates; therefore, PCR duplicates usually need to be marked or removed to keep only one copy of their original template, through deduplication. However, high-throughput sequencing has a relatively higher sequencing error rate in comparison to traditional Sanger sequencing, which poses challenges for data analysis, including deduplication. High-throughput sequencers use base quality score (*Q* score) to represent their confidence in the identity of each base [4]. *Q* scores are logarithmically related to the base calling error probabilities *P*, such that *Q* = -10 × log_10_*P* [5]. For example, a *Q* score of 10 represents an estimated sequencing error rate of 10%, and a *Q* score of 20 represents an error rate of 1%. Due to Illumina sequencing chemistry, the average base quality usually decreases from 5′-end to 3′-end of the reads [6]. Low-quality bases, often considered as bases whose *Q* scores are below 10 or 20, can be conventionally converted to the ambiguous base ‘N’s [7, 8]. Low-quality reads with too many ‘N’s are often discarded as a means of quality control [7]. As described below, the presence of ambiguous ‘N’s in the remaining sequencing reads complicates the deduplication process.

Several bioinformatics tools have been developed for deduplication which work at two distinct levels, the level of alignment results and the level of raw reads. Tools that work on sequencing alignment files include samtools-rmdup [3], Picard-MarkDuplicates [9], EAGER-DeDup [10], and gencore [11]. A common deduplication strategy of alignment-result-based tools is to drop the reads that have the same coordinates of read alignment, sometimes ignoring the underlying sequences or ambiguous bases, ‘N’s. Our previous bioinformatics pipeline for LAM-HTGTS also performs deduplication according to the alignment coordinates without considering the underlying sequences [12]. On the other hand, tools that work at the level of raw sequencing data usually perform sequence comparisons and store the unique, deduplicated sequences in a hash data structure. Such tools include pRESTO [13], clumpify [14], and dedupe [15]. However, most sequence-based deduplication tools cannot handle ambiguous base ‘N’s correctly. In order to use hash for exact sequence matching, which is efficient when handling a large amount of data, they treat ‘N’ as a different base from regular bases ‘A’, ‘C’, ‘G’, ‘T’. Hence, sequence-based tools routinely consider two reads that only differ at positions of low sequencing quality as two distinct reads. The only exception is the tool, pRESTO. pRESTO uses hash to store deduplicated sequences, and its implementation of a pairwise comparison algorithm can handle ambiguous base ‘N’s when comparing a query sequence to the stored deduplicated sequences one-by-one. However, pairwise comparison has a complexity of approximately O(n^2^), and may not be feasible due to the long processing time when dealing with large amounts of input sequences.

A major application of NGS is the repertoire analysis of antibodies in humans and animals. The analysis provides valuable information about antibody diversity and enables the identification of antibodies that have important specific functions. The variable regions of antibodies or the corresponding B cell receptors (BCRs) are encoded by V exons that are assembled by V(D)J recombination during B cell development. The most diverse part of the antibody variable region is the complementarity determining region 3 (CDR3), which includes the junctions of V–D and D–J joints for the immunoglobulin heavy chain (IgH) and V–J join for the immunoglobulin light chain (IgL). To characterize antibody repertoires, we have developed the high-throughput genome-wide translocation sequencing-adapted repertoire and somatic hypermutation sequencing (HTGTS-Rep-SHM-Seq) assay, which can cover nearly full-length of the V(D)J-rearranged sequences after merging paired-end long-length MiSeq reads [2]. This assay utilizes the genomic DNA sequence in B cells, with primers designed to target upstream of V segments and downstream of J segments, enabling characterization of the V(D)J recombination and CDR3 sequences of BCR. Each B cell has only one productive V(D)J rearranged allele for heavy chain or light chain; therefore, after deduplication, each read of productive V(D)J rearrangement will represent one B cell. Due to the high error rate of next-generation sequencing technology and the distance from the primers to the CDR3 region, a proportion of the reads capturing CDR3 sequences may contain low-quality ambiguous bases, represented as ‘N’s. Low-quality reads with too many ‘N’s are often discarded as a means of quality control [7]. Nonetheless, outright discarding sequences containing any ‘N’s carries the potential risk of overlooking some rare events, which may still be important for biological functional studies. A case in point is a kind of rare IgL that contains a rare 5-amino acid (aa) CDR3. Such IgL with 5-aa CDR3 is a conserved and functionally important feature for a type of broadly neutralizing antibody (VRC01) against HIV-1 [16]. To test our method, we analyzed the repertoire data of a mouse model that is engineered to express VRC01 class antibodies; such mouse model is used to test vaccine candidates for eliciting this kind of antibodies. Before the immunization study, it is important to determine the frequency of IgL with 5-aa CDR3, a pre-requisite for VRC01 antibody induction. By analyzing the relevant public dataset GSE214884, we observed that 5–10% of the CDR3 sequences contain ‘N’s, which increased to 12–38% for CDR3 longer than 12 aa (Additional file 1: Table S1). We observed reads that were otherwise identical except for a few low-quality bases or ambiguous ‘N’s (Additional file 1: Table S2), likely representing duplicates of the same template, although it is theoretically possible that these similar reads are indeed from different templates. An exact-matching approach to deduplication, which treats ‘N’s as distinct from other nucleotides, may artificially inflate the count of unique CDR3 sequences (Additional file 1: Table S3). For efficiency in processing huge amounts of sequencing data, our previous pipeline for HTGTS-Rep-SHM-Seq uses an alignment-result-based approach. It deals with ‘N’s by separating reads with and without ‘N’s, aligning reads with ‘N’s to reads without any ‘N’s using bowtie2, and checking their alignment length for deduplication [2]. However, this approach cannot deduplicate among reads with ‘N’s when they do not have common equivalent reads without any ‘N’s. On the other hand, by pairwise comparison, pRESTO can deduplicate among reads with ‘N’s; but it runs slowly with the tremendous amount of input sequences.

Here, we designed and implemented TrieDedup, a faster deduplication algorithm that uses the trie (prefix tree) structure to store deduplicated sequences and efficiently deduplicates at the level of raw sequences, ignoring differences only due to low-quality ambiguous base ‘N’s. We implemented a custom Python class, restrictedDict, to reduce memory usage. We benchmarked the performance of TrieDedup and the pairwise comparison algorithm implemented in pRESTO with simulated data as well as real public data. The source code of TrieDedup is available at https://github.com/lolrenceH/TrieDedup under the Apache 2.0 license.

## Implementation

### Deduplication algorithm

Many sequence-based deduplication tools regard the ambiguous ‘N’ as different from the traditional bases, ‘A’, ‘C’, ‘G’, ‘T’, using hash-based exact matching to perform deduplication. The hash algorithm is highly efficient for comparing the literal identity of sequences. However, it offers no room for correctly accounting for sequencing ambiguity. Ambiguous ‘N’s potentially represent any of the four regular DNA bases. They should not be considered as different from other DNA bases by default.

Accounting for ‘N’s in deduplication poses two challenges: (1) when allowing differences at ‘N’s, the equivalence relationship between sequences may become complicated; and (2) we need an efficient algorithm to compare between a large amount of sequences and ignore ‘N’ differences.

For Challenge (1), theoretically, a network graph of equivalence relationship can be constructed, where each node represents an input sequence, and equivalent nodes are connected by an edge. Deduplication can be regarded as the well-known 'maximal independent set (MIS)' problem on the graph. A MIS is a set of nodes that are not adjacent, and its members and their neighbors include all the nodes in the graph. A deduplicated set is equivalent to a MIS on the network graph. The complication is that MIS may be not unique, and the sizes of MISs may vary. As a toy example, a simple equivalence graph ‘TAC’–‘TNC’–‘TGC’ has an MIS {‘TAC’, ‘TGC’} and another MIS {‘TNC’}. More generally, a star-shaped graph can have an MIS consisting of the tip nodes, or another MIS consisting of the center node. Thus, we need a principle for choosing a MIS. For sequence deduplication, we may prefer to choose the nodes with fewer ‘N’s to represent the observed sequences, which correspond to the tip nodes of the star-shaped graph.

**Fig. 1:**
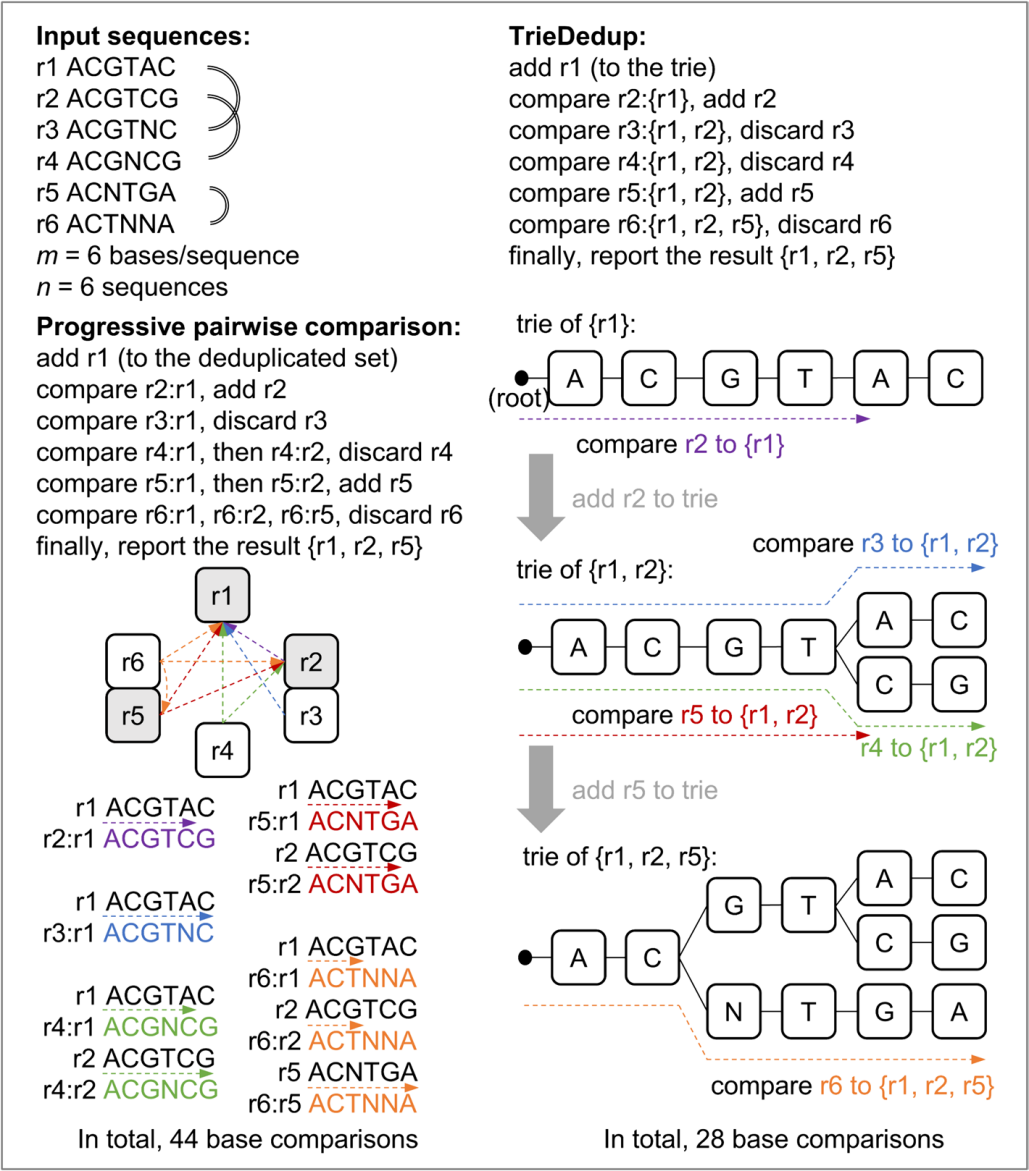
Diagram of progressive pairwise comparison and TrieDedup algorithm

Finding a MIS can be achieved by adding a candidate node into a MIS and removing neighbors of the node from the query, iteratively. Instead of performing a pairwise comparison between all input sequences, we can store unique sequences that are previously deduplicated and compare each query sequence to these established deduplicated sequences, reducing the number of comparisons (Fig. 1). Because we prefer unambiguous sequences, we sort the input sequences by the number of ‘N’s in ascending order, and consider each read, sequentially. This progressive pairwise comparison is implemented in pRESTO.

For Challenge (2), the pairwise comparison algorithm needs to compare each input sequence with the full set of deduplicated sequences to determine if it is unique. We adapted the trie (prefix tree) structure to store the previously deduplicated sequences, whose prefixes are organized into a consensus tree. The trie structure can retain information of sequence similarity from previous comparisons, thereby reducing the number of necessary comparisons. The trie structure can immediately identify an unobserved sequence, as soon as the input sequence diverges from the observed paths, thus reducing the number of comparisons (Fig. 1).

In summary, we designed and implemented the following algorithms to store and compare sequences, which can ignore mismatches due to ‘N’s.

**Algorithm 1:**
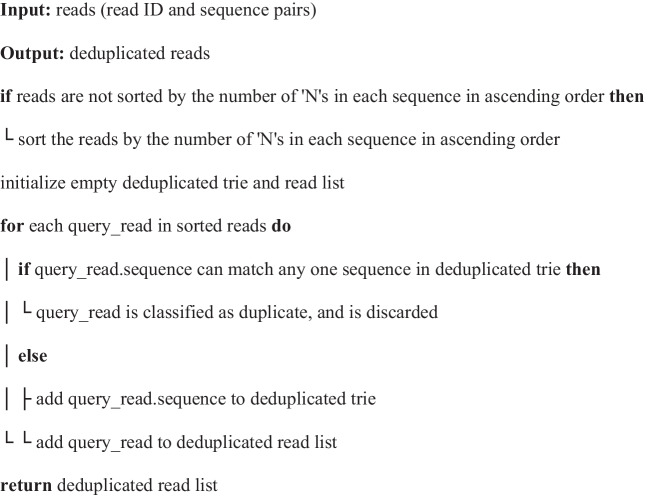
Deduplication with trie storing a working set of deduplicated sequences

**Algorithm 2:**
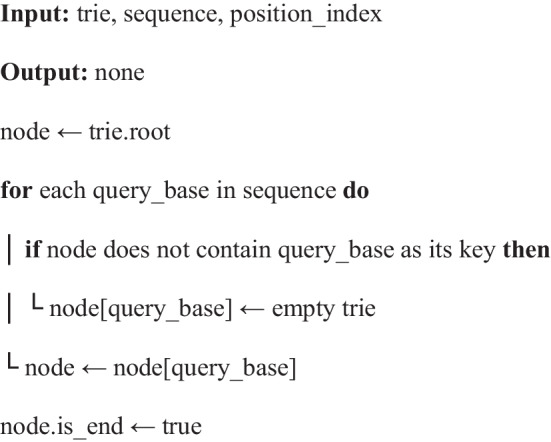
Adding a sequence to the trie storing already deduplicated sequences

**Algorithm 3:**
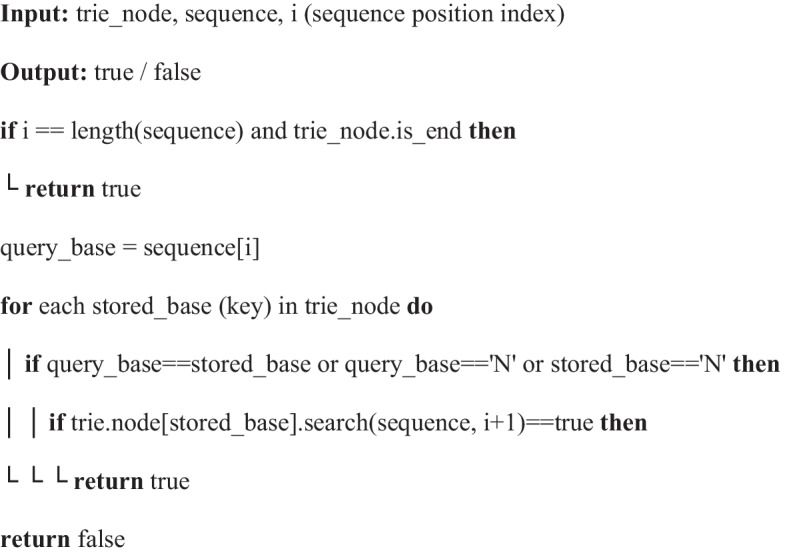
Searching for a query sequence in the trie storing already deduplicated sequences

Note:

1. keep ‘N’ the last stored_base (after regular bases) when traversing trie_node
2. searching the whole sequence from trie_root by: trie_root.search(sequence, 0)

As an implementation detail, to avoid repeatedly checking the same sequence, we also implemented exact-matching deduplication using a hash dict. This step is executed before performing actual deduplication through the trie algorithm. Subsequently, sequences are sorted in ascending order based on the number of ‘N’s present, ensuring that high-quality sequences are processed first, recognized as unique, and stored in the already deduplicated set. Sequences with more ‘N’s are processed later and progressively matched against those deduplicated sequences with fewer ambiguous ‘N’s.

### Memory usage optimization

The pairwise comparison algorithm directly stores every deduplicated, unique sequence, while our algorithm stores them in the trie structure, which theoretically costs less memory. However, empirically, storing a trie structure may cost more memory on the dict data structure than just storing simple sequences in hash keys, potentially due to Python’s base-level optimizations. To reduce memory consumption, we used __slots__ magic in Python to declare the variables. In Python, by default, a class instance declares a dict attribute named __dict__ to dynamically store its variable names and values. The __slots__ attribute in Python allows for the declaration of a fixed set of attributes for class objects, preventing the creation of the instance dict __dict__, thus significantly reducing the memory footprint of each class instance. We also implemented restrictedDict, a restricted dict with list implementation: it restricts hash keys to predetermined options, stores the values in a list instead of a dict, and keeps a shared dict to map limited keys to the index in the list. The restrictedDict achieves a smaller memory usage by storing values in a list instead of a dict, accommodating for large sequencing datasets. In DNA sequencing data, there are five possible base identities: 'A', 'C', 'G', 'T', and ‘N’. Thus, we only keep one dict as a shared class variable of restrictedDict, which stores the mapping table from the base letter (as the keys of dict) to the index in the list. For simplicity in describing the results, we abbreviate the original trie implementation as trie0, trie with only __slots__ magic as trie1, and trie with __slots__ and restrictedDict as trie2.

### Benchmark and comparison to other tools

We compared progressive pairwise comparison algorithm and trie implementation with or without memory optimization in a Dell PowerEdge T640 server, with 192GB physical memory and limited to one CPU of Intel Xeon Gold 6126 2.6GHz, and in RedHat Linux operation system. In order to ensure a fair comparison with pRESTO, which is implemented in Python and has additional functions that may affect its efficiency, we reimplemented the progressive pairwise comparison algorithm according to pRESTO. We benchmarked the accuracy, running time, and memory usage of the exact-matching, pairwise comparison, and trie algorithms using both simulated reads or real public reads as input.

To simulate input sequences, we first generated a unique set of parental sequences of a specific length, then randomly sampled them with replacement, which usually sampled approximately 55% of unique parental sequences, and lastly converted a fixed number of bases at random locations to ‘N’s in each sampled sequence. We considered sequences originating from the same parental sequence to be PCR duplicates; thereby we knew the ground truth for the size of the deduplicated set. We performed comparisons for 10^3^, 2 × 10^3^, 5 × 10^3^, 10^4^, 2 × 10^4^, 5 × 10^4^, 10^5^, 2 × 10^5^, 5 × 10^5^, and 10^6^ input sequences, with lengths of 30, 100, 150, and 200 bp, and converted 1%, 5%, 10% or 20% bases to ‘N’s for each input sequence. Each condition is tested with three repeats.

We also benchmarked the performance in public HTGTS-Rep-SHM-seq data of the BCR repertoire. We downloaded raw fastq files for SRR3744758, SRR3744760 and SRR3744762 from SRA, each containing slightly more than 1 million 300-bp paired-end reads. We then randomly selected 1 million reads and masked bases with a quality score ≤ 10 by ‘N’ to serve as the input sequences.

## Results

### Theoretical complexity analysis

Suppose there are *n* input sequences, and each sequence has *m* bases. For the preprocessing steps, the time complexity of counting ‘N’s is O(*m* × *n*), and sorting *n* sequences can be O(*n* × log(*n*)) for quick sort, or O(*n*) for bucket sort.

Suppose we progressively add sequences to the deduplicated set, and the deduplicated set has already stored *n*_d_ sequences. The space complexity of plain storing *n*_d_ sequences is O(*m* × *n*_d_). The algorithm of pairwise comparison between a candidate sequence and the deduplicated set has time complexity O(*m* × *n*_d_). Then, the overall complexity of the whole deduplication process is O(*m* × *n*^2^). This algorithm has been implemented in pRESTO, which runs slowly with a large number *n* of input sequences.

Here, we designed and implemented an algorithm using the trie structure to store the already deduplicated sequences. The upper limit of the space complexity of the trie structure storing *n*_d_ sequences is O(*m* × *n*_d_), and should be lower when the sequences have common prefixes. Without ‘N’s, the time complexity of comparison between a query sequence and the trie structure is only O(*m*); therefore, the lowest overall complexity of the whole deduplication process is O(*m* × *n*). However, when allowing ambiguous base ‘N’s, we may need to explore more branches in the trie to determine the comparison result. Theoretically, the upper limit of complexity for one query sequence is O(*m* × *n*_d_); therefore, the upper bound of the overall complexity is still O(*m* × *n*^2^). Though, this situation may rarely happen as long as the input sequences do not contain too many ‘N’s. If each sequence has at most *k* ‘N’s, the upper limit of the time complexity between one query sequence and the trie structure is O(*m* × 5^*k*^), for 5 possible choices of bases ('A', 'C', 'G', 'T', ‘N’) at *k* trie nodes; therefore, the overall time complexity is O(*m* × *n* × 5^*k*^). Theoretically, the actual time complexity is dependent on the amount and location of ‘N’s in sequences. Sequences with fewer ‘N’s or ‘N’s located closer to the end (near trie tips) will have less complexity than those with more ‘N’s or ‘N’s located closer to the start (near trie root).

### Benchmark on simulated data

We benchmarked the accuracy, speed and memory consumption of the trie algorithm with or without memory optimization, and compared them to the performance of the progressive pairwise comparison algorithm, which we reimplemented from pRESTO, using the simulated 200-bp reads. Both the pairwise comparison and trie algorithms demonstrate high accuracy in recovering the deduplicated sets to the ground truth sizes (Additional file 1: Table S4). In contrast, the exact-matching approach, which treats ‘N’s as distinct from other nucleotides, inflates the sizes a lot (Additional file 1: Table S4), although it runs very fast (< 2.5 s for *n* = 10^6^) and requires minimal memory (< 0.7 GB for *n* = 10^6^).

**Fig. 2:**
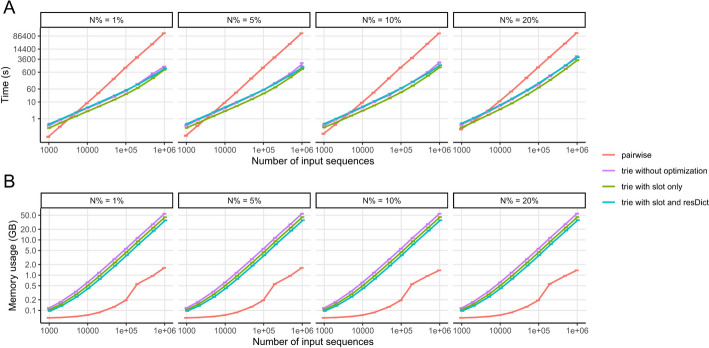
Running time and memory usage increases with larger amount of input sequences (benchmark simulation). **A** Running time; **B** memory usage. Input sequences are 200 bp in length. Error bars shows mean ± standard deviation, each with 3 replicates

The pairwise comparison and all the trie algorithms show an approximately linear relationship between log-transformed running time and the log-transformed number of input sequences (Fig. 2A). The slope of pairwise comparison is close to the theoretical order 2. On the other hand, the slope of the trie algorithm is much lower, which is ≤ 1.3 for *n* ≤ 10^5^ and the percentage of ambiguous bases in reads (N%) ≤ 10%, and increases for larger *n* or N%. When the input sequences are less than 5000, the pairwise comparison algorithm is more efficient than the trie algorithm whose performance is less than 3s. When there are more than 5000 input sequences, the trie algorithm runs significantly faster than pairwise comparison.

The trie algorithm significantly outperforms pairwise comparison at large input sizes. For example, at N% = 5% and *n* = 10^4^, pairwise comparison needs 8.9s (on average), trie algorithm needs ≤ 5.0s; at *n* = 10^5^, pairwise comparison needs 1100 s (or 18.3 min), trie algorithm needs ≤ 55s; at *n* = 10^6^, pairwise comparison needs 125000s (or 34.7h), trie algorithm needs ≤ 1755s (or 29.3 min).

When comparing the running time among the three different options of memory optimization of the trie implementation, we found that all the three options have very similar running time, without magnitude difference. Trie1 runs relatively faster than trie0 and trie2, with the difference being less than twofold (Fig. 2A).

In terms of memory usage, the pairwise algorithm is more memory-efficient to implement than the trie because entire sequences can be stored as one item instead of storing each base individually (Fig. 2B). Among three trie implementations, as expected, trie0 requires the most memory and trie2 requires the least. The slopes for the three options in Python implementation are similar, all close to but a little less than the theoretical 1. For *n* ≥ 10^5^, the implementation of restrictedDict (trie2) improves the memory usage of __slots__ optimization (trie1) by approximately 19%, while trie1 improves 20% compared to trie0; therefore, trie2 only uses 65% memory as much as trie0 uses.

When N% = 5% and *n* = 10^4^, pairwise comparison on average requires 0.07GB memory to run, while trie0 requires 0.62GB, trie1 0.51GB, and trie2 0.42GB; when *n* = 10^5^, pairwise comparison needs 0.19GB, trie0 5.6GB, trie1 4.5GB, and trie2 3.7GB; when *n* = 10^6^, pairwise comparison requires 1.6GB, trie0 55GB, trie1 44GB, and trie2 36GB.

We also evaluated the influence of the length of input sequences and N% on the performance of pairwise comparison and trie2 (Additional file 2: Fig. S1, Additional file 2: Fig. S2). As expected, longer length will need more running time and memory usage. The percentage of ambiguous bases in reads (N%) barely impacts the memory usage of both algorithms. This is likely because the memory usage is more closely correlated with the number of unique reads stored in the already deduplicated set, whose ground truth remained constant across different N% in our simulation. On the other hand, a higher N% increases the running time of the trie algorithm, while it does not affect the running time of pairwise comparison. This may be because the pairwise comparison algorithm requires a lot of comparisons between sequence pairs until it finds a match, which is not significantly affected by N; whereas the trie algorithm needs to search through more branches when the query sequence contains more ‘N’s. Additionally, when a sequence with more ‘N’s is considered unique and added to the trie structure, it may slow down subsequent searches by increasing the possible branches at ‘N’.

### Benchmark on real data

**Fig. 3:**
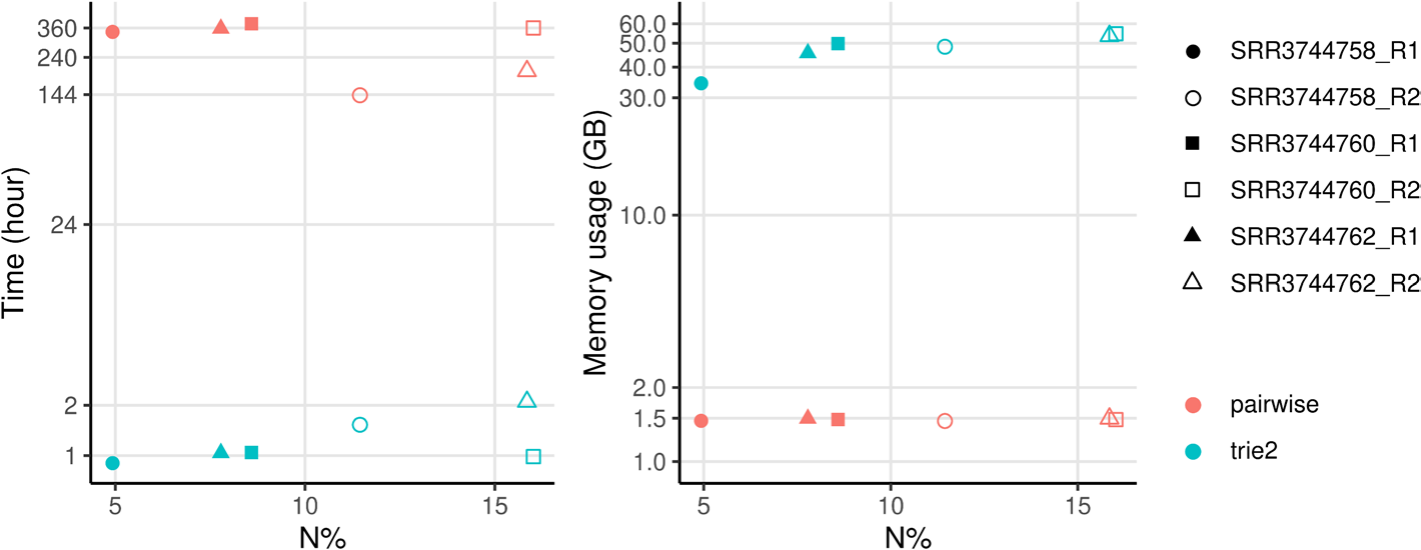
Running time and memory usage when applying on 10^6^ real 300-bp reads from SRA. X-axis ‘N%’ shows the average percentage of ambiguous base ‘N’s in reads

We applied the exact-matching, pairwise comparison, and trie algorithms to published sequencing data with a read length of 300 bp and an average N% of 4.9–15.8%. As expected, higher N% and a lower percentage of unique reads were observed in R2 than in R1 reads. With an input size of 10^6^ raw reads, both pairwise comparison and trie algorithms reported the same numbers of unique reads (Additional file 1: Table S4). In contrast, although the exact-matching approach runs very fast (< 4 s) and requires minimal memory (< 0.75 GB), it likely inflates the sizes of deduplicated sets, especially for R2 reads, whose N% is higher than R1 reads (Additional file 1: Table S4). Deduplication by trie2 only needed 0.9–2.1 h using 35–55 GB of memory, while deduplication by pairwise comparison required 6–16 days with about 1.5 GB memory usage (Fig. 3). Therefore, trie2 deduplication can achieve about 270-fold faster speed than pairwise comparison, with 32-fold higher memory usage.

## Discussion

To deduplicate high-throughput sequencing libraries while ignoring differences only due to ambiguous base ‘N’s, we adapted the trie structure to store deduplicated sequences, and implemented a corresponding algorithm, named TrieDedup. When the input size is larger than 5000 sequences, the trie algorithm is more efficient than the pairwise comparison algorithm used in pRESTO, at the price of higher memory usage. TrieDedup can deduplicate up to 10^6^ input sequences within 2 h using less than 36GB of memory. In addition, TrieDedup may be potentially adapted into pRESTO framework.

The real data we used for benchmarking were public HTGTS-Rep-SHM-seq data of BCR repertoire downloaded from SRA (SRR3744758, SRR3744760, and SRR3744762). For such a typical study targeting B cell receptor repertoire, these samples originally contain 1.22 million, 1.10 million, and 1.49 million 300-bp reads respectively, with the fastq file sizes being approximately 1.6 GB, 1.5 GB, and 2 GB. For our benchmark experiments, we randomly selected 1 million reads from these samples, believing that this number represents a realistic read number for this type of sequencing data. For the other genomic studies, the read number can vary significantly in scale, which depends on the targeted genomic region size, the depth of sequencing, and the lengths of sequenced reads. For instance, a 30X Whole Genome Sequencing (WGS) of a human genome typically requires around 900 million 100-bp reads, while an 80X Whole Exome Sequencing (WES) might need about 24 million 100-bp reads. These quantities are 1–3 orders of magnitude higher than those typical targeted sequencing such as B cell repertoire studies. Our benchmark suggests a dataset of up to 1 million reads, or a fastq file size of approximately 1.5GB, can be efficiently deduplicated by our TrieDedup with approximately 36GB of memory usage which a typical server has the memory capacity to support. Since both the running time and memory usage increase with the number of input sequences, and the complexity order of running time on N exceeds one, the efficiency of deduplication may be further improved if we can divide the input sequences into smaller non-overlapping groups. To handle a larger number of reads, we also recommend grouping reads to reduce each group to below 1 million reads. For example, the reads may be grouped based on sequence prefixes, or the mapped chromosome and coordinate range if the reads have been aligned, or the V and J alignment for V(D)J repertoire. If these subsets of data are processed separately, the algorithm will have to store fewer reads simultaneously, hence reducing memory usage.

Trie structure has also been used in the deduplication of Unique Molecular Identifiers (UMIs) [17], but the traditional trie structure cannot handle ambiguous bases, although errors in UMIs are common [18]. UMIs containing any ‘N’s or bases with a *Q* score below 10 are by default filtered out during 10x Genomics Cell Ranger processing. Here, we designed and implemented TrieDedup, where the specialized trie structure and algorithm can correctly and efficiently handle the differences due to ambiguous bases. With its ultra-fast algorithm, TrieDedup may also potentially be applied to barcode or UMI assignment when considering reads with a few low-quality bases in the UMIs.

We designed, implemented, and showcased a universal algorithm using a trie for deduplication that allows for ambiguous letter matching, therefore, we did not restrict the allowed keys of restrictedDict to ‘A’, ‘C’, ‘G’, ‘T’ and ‘N’. Our highly versatile TrieDedup algorithm can be applied not only to DNA reads, as evaluated in the manuscript, but also directly to protein amino acid sequences and even text word matching. To further improve runtime and memory usage, we also implemented TrieDedup in Java and C++, but hard-coded the allowed keys to DNA bases. These implementations are also available in our GitHub repository. Compared to Python implementation, TrieDedup C++ implementation achieved 5–11-fold faster and 1/3 memory. However, it imposes limitations by being restricted solely to DNA sequences, underscoring a trade-off between performance optimization and general applicability.

The threshold of *Q* scores for converting low-quality bases to ambiguous ‘N’s, which is often library-specifically set to 10 or 20 arbitrarily, may affect N% in input reads, as well as the amount of deduplicated reads. A potentially more principled approach is to sum up the error rate of mismatches in pairwise comparison, and then set the threshold on the sum error rate to judge the equivalence between reads. However, it may generate a more complicated relationship of equivalence and even higher computational complexity than the current pairwise comparison algorithm in pRESTO.

## Conclusions

We implemented TrieDedup, which uses the trie structure to store deduplicated sequences, and ignores differences only due to ambiguous base ‘N’s. We also implemented a memory-efficient class, restrictedDict, that reduced the memory usage to about 0.8-fold. TrieDedup significantly outperforms the pairwise comparison strategy when the amount of input sequences is larger than a few thousand. TrieDedup can deduplicate reads up to 270-fold faster than pairwise comparison at a cost of 32-fold higher memory usage. Potentially, TrieDedup may be adapted into pRESTO, and may be generalized to other scenarios for deduplication with ambiguous letters.

### Supplementary Information

**Additional file 1: Table S1.** The percentage of CDR3 sequences containing ambiguous bases (‘N’s) in public dataset GSE214884. **Table S2.** Unique and potential duplicate sequences with ambiguous bases ‘N’s in 5-aa CDR3 sequences from GSM6617404. **Table S3.** The number of CDR3 sequences before and after deduplication in public dataset GSE214884. **Table S4.** The number of sequences after deduplication for benchmark analysis.

**Additional file 2: Figure S1.** Running time for input sequences with different lengths. Error bars shows mean ± standard deviation, each with 3 replicates. **Figure S2.** Memory usage for input sequences with different lengths. Error bars shows mean ± standard deviation, each with 3 replicates.

## Data Availability

TrieDedup code is available at https://github.com/lolrenceH/TrieDedup. The real data we used for benchmarking are public data downloaded from NCBI-SRA, with accession number: SRR3744758 (https://www.ncbi.nlm.nih.gov/sra/SRR3744758), SRR3744760 (https://www.ncbi.nlm.nih.gov/sra/SRR3744760) and SRR3744762 (https://www.ncbi.nlm.nih.gov/sra/SRR3744762). Project name: TrieDedup: A fast trie-based deduplication algorithm to handle ambiguous bases in high-throughput sequencing. Project home page: https://github.com/lolrenceH/TrieDedup. Operating system(s): Platform independent. Programming language: Python, with C++ and Java implementations available on GitHub. Other requirements: seqtk, pandas. License: Apache 2.0. Any restrictions to use by non-academics: None.

## Abbreviations

PCR: Polymerase chain reaction
*Q* score: Base quality score
BCR: B cell receptor
CDR: Complementarity-determining region
HTGTS-Rep-SHM-Seq: High-throughput genome-wide translocation sequencing-adapted repertoire and somatic hypermutation sequencing
aa: Amino acid
MIS: Maximal independent set
trie0: Trie implementation without memory optimization
trie1: Trie implementation with only __slots__ magic
trie2: Trie implementation with __slots__ and restrictedDict
N%: Percentage of ambiguous bases in reads
UMIs: Unique Molecular Identifiers
