# Supplementary material for: TrieDedup: a fast trie-based deduplication algorithm to handle ambiguous bases in high-throughput sequencing
Source: BMC Bioinformatics. 2024 Apr 18;25:154. doi: 10.1186/s12859-024-05775-w (PMC11025179; doi:10.1186/s12859-024-05775-w)
Supplement: Supplementary file 2 — Additional file 2: Figure S1. Running time for input sequences with different lengths. Error bars shows mean ± standard deviation, each with 3 replicates. Figure S2. Memory usage for input sequences with different lengths. Error bars shows mean ± standard deviation, each with 3 replicates. [file 12859_2024_5775_MOESM2_ESM.docx]

**Figure S1. Running time for input sequences with different lengths.** Error bars shows mean ± standard deviation, each with 3 replicates.

**Figure S2. Memory usage for input sequences with different lengths.** Error bars shows mean ± standard deviation, each with 3 replicates.
